# Single molecules can operate as primitive biological sensors, switches and oscillators

**DOI:** 10.1186/s12918-018-0596-4

**Published:** 2018-06-18

**Authors:** Rosa D. Hernansaiz-Ballesteros, Luca Cardelli, Attila Csikász-Nagy

**Affiliations:** 1 Randall Centre for Cell and Molecular Biophysics and Institute for Mathematical and Molecular Biomedicine, King’s College London, London, SE1 1UL UK; 2 Microsoft Research, 21 Station Road, Cambridge, CB1 2FB UK; 3 Department of Computer Science, University of Oxford, Wolfson Building, Parks Road, Oxford, OX1 3QD UK; 4 Faculty of Information Technology and Bionics, Pázmány Péter Catholic University, Budapest, H-1083 Hungary

**Keywords:** Computational biology, Mathematical modelling, Multistability, Bistability, Oscillation, Networks, Circadian rhythm, RNA world, Evolution, Approximate majority

## Abstract

**Background:**

Switch-like and oscillatory dynamical systems are widely observed in biology. We investigate the simplest biological switch that is composed of a single molecule that can be autocatalytically converted between two opposing activity forms. We test how this simple network can keep its switching behaviour under perturbations in the system.

**Results:**

We show that this molecule can work as a robust bistable system, even for alterations in the reactions that drive the switching between various conformations. We propose that this single molecule system could work as a primitive biological sensor and show by steady state analysis of a mathematical model of the system that it could switch between possible states for changes in environmental signals. Particularly, we show that a single molecule phosphorylation-dephosphorylation switch could work as a nucleotide or energy sensor. We also notice that a given set of reductions in the reaction network can lead to the emergence of oscillatory behaviour.

**Conclusions:**

We propose that evolution could have converted this switch into a single molecule oscillator, which could have been used as a primitive timekeeper. We discuss how the structure of the simplest known circadian clock regulatory system, found in cyanobacteria, resembles the proposed single molecule oscillator. Besides, we speculate if such minimal systems could have existed in an RNA world.

**Electronic supplementary material:**

The online version of this article (10.1186/s12918-018-0596-4) contains supplementary material, which is available to authorized users.

## Background

**Fig. 1:**
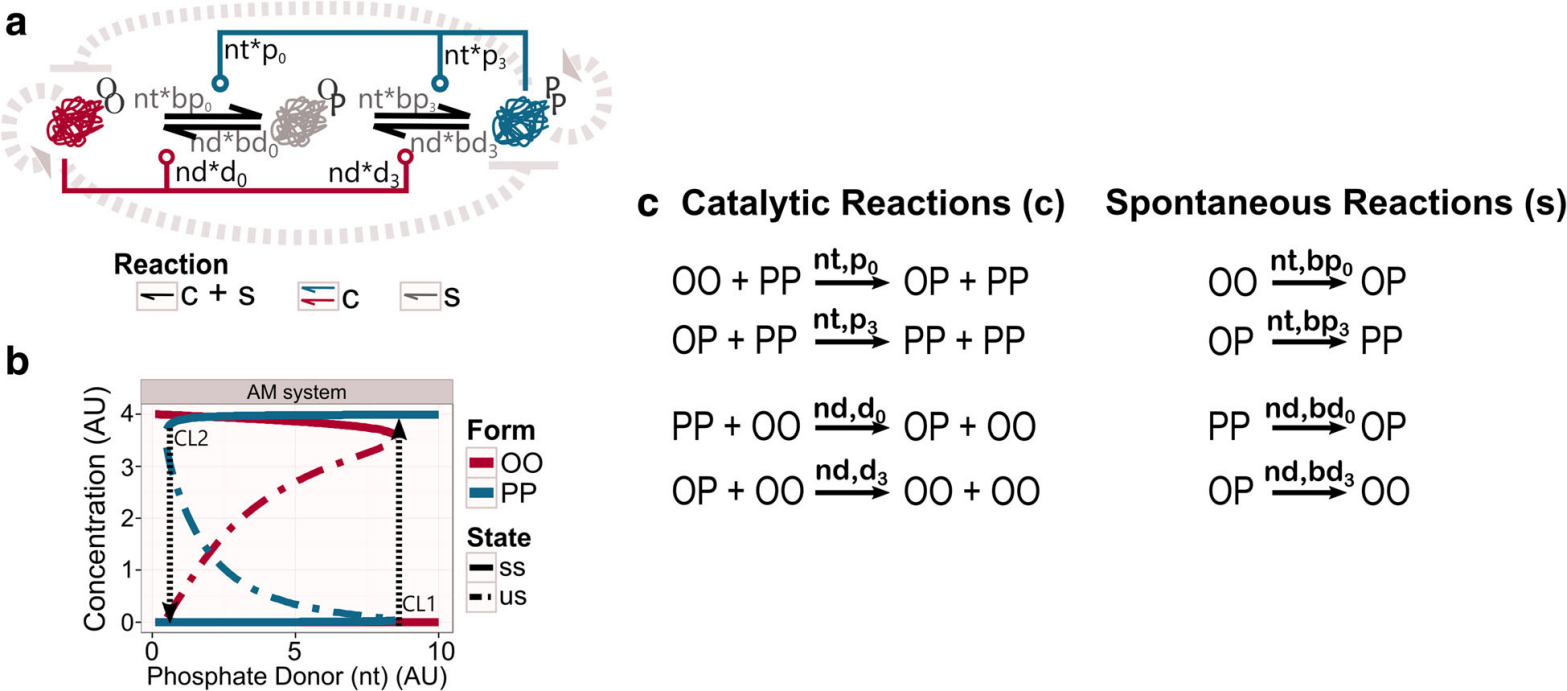
Approximate Majority (AM) system. **a** Wiring diagram of the AM network. PP form attracts molecules into this state, while OO form does the opposite, both via catalytic reactions. Background reactions happen between all conformations at a low rate (grey shades on arrows). As an illustration of the embedded positive feedback loops, striped grey arrows indicate the pure positive feedback loops. The striped dash-end indicate the double-negative positive feedback loop. **b** Bifurcation analysis of the AM system. The plot shows how the concentration of the catalytic molecules, OO and PP, is affected by the availability of phosphate donor (nt). The stable steady state (ss) is indicated by a solid line, while the unstable steady state (us) is defined by the dash-dotted line. CL1 and CL2 indicate the threshold points at which the jump between the *off* and the *on* states happen. Black striped arrows indicate the direction of the jumps. See *networks topology* in Methods for more information about the structure. Consult Table 2 in Methods for values of each parameter. **c** List of reactions. The left column shows the catalytic reactions, while the right column presents the spontaneous reactions. On top of each reaction arrows, the parameter names that affect the specific reaction are indicated

Molecular networks exhibit a variety of dynamical behaviours based on the necessities of the cell [1–3]. Switch-like dynamics, such as toggle-switches, are usually found in regulatory processes of decision-making systems, as the system needs an all-or-none response. Toggle-switches transform a gradient input signal into an *on/off* output [4–7]. Changes in the input level cause that the responding molecule switches between low and high activities and/or concentrations (i.e. between *off* and *on* states). These changes cause shifts in the dynamical stability of the response, so the steady states of the system can be followed on a bifurcation diagram or signal-response curve [3, 8, 9]. Due to the nonlinear nature of the toggle-switch, if the signal is below a critical level (CL1), the response will be kept in one state (i.e. low concentration or *off* state); when the signal increases enough to go through this critical level, the response will abruptly change to the other state (i.e. high concentration or *on* state) (Fig. 1). For intermediary values of the signal, a bistable regime may arise. Over this region, the state of the response is not determined solely on the signal value, but it also depends on the earlier state of the response. Thus, an increase of the signal allows the system to pass the CL1 and reach the *on* state. At this stage, the system will not be able to go back to its previous state, *off* state, until the signal decreases enough to pass another, lower, critical level (CL2) [4–6, 10, 11] (Fig. 1).

Biological systems present remarkably nonlinear dynamical features. A typical highly non-linear mechanism is the multi-step modification of proteins, like phosphorylation, methylation or ubiquitination [12, 13]. Consider a molecular population where most molecules are in the same *off* state (i.e. dephosphorylated, unmethylated, etc.) (below CL1, Fig. 1b). In this stage, the molecular population can repeatedly bind activators (i.e. phosphate donors in the case of phosphorylation) to be modified on a single site at each encounter (distributive multistep modification), without changing the overall *off* state response. The active or *on* state will be abruptly achieved when the molecules get modified at a critical ratio or critical site. This critical event leads to a major conformational change that will activate them (above CL1; Fig. 1b).

Responding molecules and their activators are embedded in positive feedback loops to coordinate a biological toggle-switch [2, 3, 7]. Positive feedback loops can contain only positive (activating) interactions between the involved molecules (pure positive feedback loop). They can also contain an even number of negative interactions (double-negative positive feedback loop) [10, 14]. Such interactions usually lead to bistability and the hysteresis effect. A bistable system offers two stable states: when the input level is below the critical level CL2, or above CL1, the system rests in a unique stable state, either the *on* or the *off* state. Between these critical levels, the system can rest in both stable states (Fig. 1b). The system will rest in either of the states depending on its previous history. Such effect is called hysteresis. Hysteresis can stabilise the system in either state even with a noisy input signal, making the system resilient to signal perturbations or noise [15].

The Approximate Majority system, a simple network first developed as an algorithm for distributed computing [16], presents these properties: nonlinearity, bistability and hysteresis [5, 6]. This network relies on three positive feedback loops (two pure positives and one double-negative) to generate both bistability and hysteresis (Fig. 1a; background grey dashed arrows indicate the implicit feedback loops of the system). The nonlinear response is obtained by successive multi-step modifications of molecules. Although these features are necessary properties for toggle-switch behaviour, the major advance of the Approximate Majority (AM) network is its efficient minimal topography. The AM system is considered as a minimal topology due to the number of molecular types that define it: a single kind of molecule that can be found in three different states. The two extreme forms hold catalytic activities for their own activation and inhibition, while an intermediate, undecided state separates them [5]. From the computing point of view, the AM systems is described as an efficient algorithm, since it is able produce a fast and robust toggle-switch by using a minimal number of components [16].

The dynamical features of the AM network have been associated with biological regulatory networks at various levels of complexity [6]; from the epigenetic cell memory system [17] to the G2/M transition of the cell cycle [5]. However, as far as our current knowledge goes, existing biological systems do not hold an exact organization of a single autocatalytic molecule that can work as an efficient toggle-switch. Earlier we have shown that AM can emulate the behaviour of much more complex biological switches and it stands as the smallest of such systems [6, 18]. In this work we would like to study how currently observed bistable biological regulatory networks could have emerged from a simple network, like AM.

**Fig. 2:**
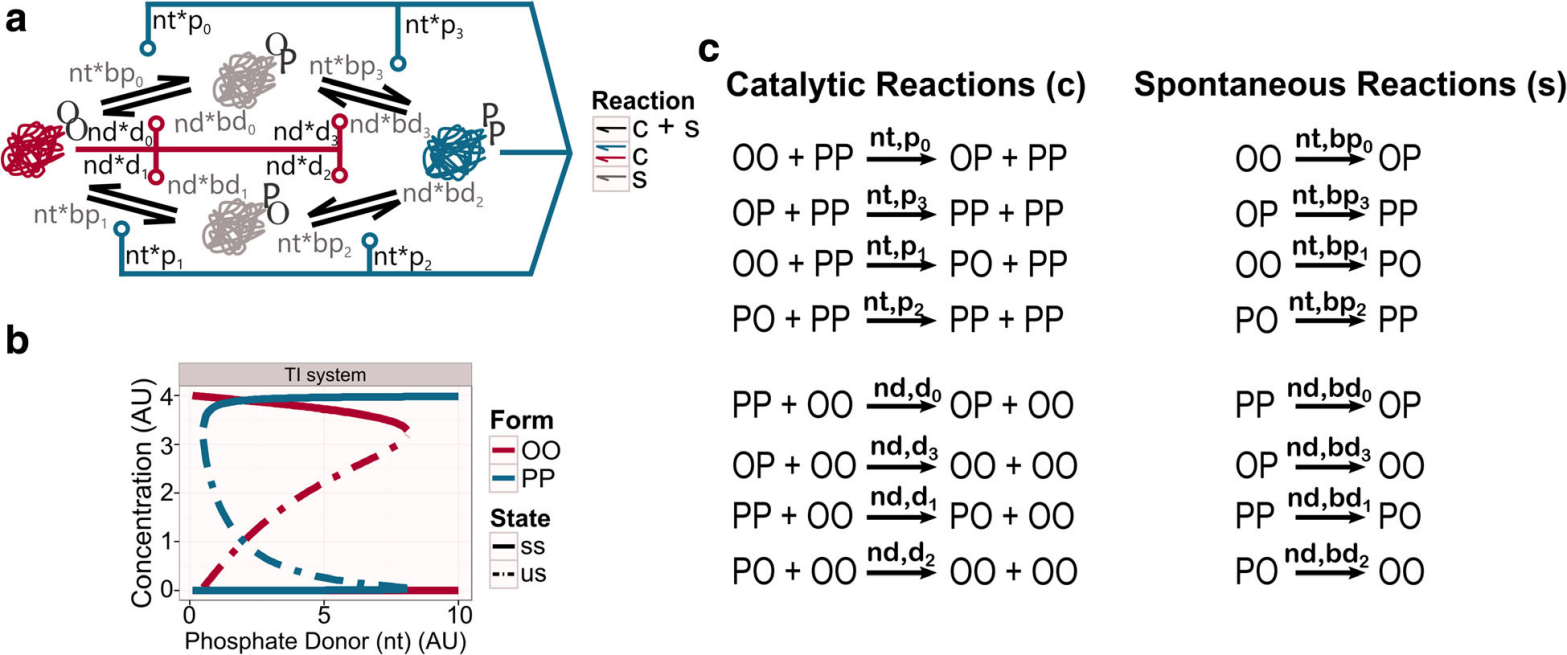
Two intermediates (TI) system. **a** Wiring diagram of the TI system. Single-modified forms (OP, PO) are physically different. **b** Bifurcation analysis of TI system. The graphic shows how the concentration of the catalytic molecules, OO and PP, is affected by the availability of phosphate donor (nt). We speculate that the TI system could have served as a primitive molecular sensor of phosphate donor level. Thus, TI could get into the PP form only above a critical threshold, where it remains until the phosphate level drops below a lower critical level. The stable steady state (ss) is indicated by a solid line, while the unstable steady state (us) is labelled by the dash-dotted line. See *networks topology* in Methods for more information about the structure. Table 2 in Methods for values of each parameter. **c** List of reactions. The left column shows the catalytic reactions, while the right column gives the spontaneous reactions. On top of each reaction arrow, the parameter names that affect the specific reaction are indicated

The AM is based on a non-biological system; to get it closer to biological examples, we go into the molecular details of what AM can represent. AM presents a single intermediate state, which from a molecular perspective, is not precise. In the more realistic *Two Intermediates* (TI) system (Fig. 2), we split the single intermediate state of AM into two intermediate forms. These forms indicate the modification pattern of the two distinct sites. Thus, TI could be considered as an ancient biological system, where a single molecule can be modified in two separate sites. These modifications change its conformation in such way that it will hold different activities in the extreme forms. The TI network could work as a primitive molecular sensor due to its toggle-switch behaviour. This minimal system could have evolved by undergoing structural modifications, altering its topology and having an impact on its dynamical function.

Under these premises, we derive reduced models from the TI system (with a basic set of parameters) and study their dynamical behaviours through steady-state analysis. We find that the toggle-switch behaviour, and therefore the function to work as a sensor, can be preserved for a large variety of topological changes. This highlights that regulatory networks derived from the AM class of systems are robust and efficient decision makers. Furthermore, we find that some topological alterations can transform the toggle-switch dynamics into an oscillatory one. This could allow the evolution of new biological functions originating from a minimal AM-like structure. We discuss two biological examples that could have emerged from a minimal system like AM. The circadian rhythm regulatory network of cyanobacteria shows high similarities to the oscillatory derivatives of the AM network. Dynamical features of riboswitches, that are also ribozymes, could also be related to the presented systems. These highlights that evolution might have found this system simple but robust, relying on these AM-like type of systems to build up some current regulatory networks.

## Results

### The two intermediates (TI) system, a realistic AM-like system working as a molecular sensor

The *Two Intermediates* (TI) network is composed of a single kind of molecule with two antagonistic autocatalytic activities. This motif is a double-site modification system where the autocatalytic states act as enzymes. This network can generate a toggle-switch behaviour due to interactions between the different forms, embedded in several positive feedback loops (Fig. 2a). Molecules of the TI network can be found in three different forms, based on the number of modifications they hold: none (OO), single (OP/PO) or double (PP). They turn between conformations either spontaneously (slow) or by a catalyst (fast). OO and PP conformations hold catalytic activities, so they are not only substrates in the reactions but catalysts (see Methods). PP forms increase the number of modifications in the other forms (pure positive feedback loop), while the catalytic form OO removes these modifications (pure positive feedback loop). Thus, each catalytic conformation attracts molecules to that specific state, and away from the other (double-negative feedback loop).

Modifications usually induce structural changes in the conformations. The addition of a modification may affect the exposure of other sites, by burying or exposing them. Molecules of the TI system present two sensitive sites of modification, and depending on which site is modified first, a different single-modified form will be obtained (either OP or PO) (Fig. 2a). Thus, two separated patterns of modification are established in the TI network. For example, to reach a double-modified form (PP) through OP, the second site (_P) is always modified first, followed by the first site (P_). To come back to the unmodified form (OO) through the same single-modified form (OP), the first site (P_) needs to be unmodified first.

From a dynamical point of view, the TI system is a suitable candidate to work as a molecular sensor, since its toggle-switch dynamics create an all-or-none response (Fig. 2b). A molecular sensor informs downstream processes about changes in the environment. Particularly, such all-or-none switch can convert an analogue change in the input into a digital (none or all) output. Considering TI as a minimal topology for sensing, the simplest way for carrying out its function is responding to changes in the molecules that affect its modifications. When the sensed compound is in excess, the molecules of TI are fully modified; if the sensed compound level is low, molecules are fully unmodified.

The TI system, as many biological processes, needs an energy supply to accomplish its function. Phosphates, and more concretely highly energetic nucleotides like NTP, were early established as energy molecules [19, 20]. Thus, the simplest way for spontaneous modifications to take place is to use the cleaved phosphate from a phosphate donor, like NTPs, to modify itself. Using the same mechanism, low energetic nucleotides, such as NDPs or NMPs, can be utilised as phosphate acceptors to remove these modifications. Equivalently, the catalytic form PP uses NTPs to add phosphates into the other forms, attracting them to the double-modified state. OO form uses low energetic nucleotides to remove these phosphates, attracting the molecules to the unmodified state. In a way, the catalytic form PP works like kinases, and the catalytic form OO as a kind of phosphatase that reverses the kinase reactions by using the same catalytic core, as it happens at some bifunctional kinases [21–23].

The concentration of free NTP, or any phosphate donor (*nt* from now on), can be sensed by AM and TI networks, as all modification reaction rates depend on the *nt* level. Thus, we consider the TI network as a feasible primitive nucleotide or phosphate sensor. Molecules are fully modified (double-phosphorylated, *PP* form) when *nt* is above a critical level; they switch back to full unmodified (dephosphorylated or *OO* form) only at a much lower *nt* level.

### The TI system preserves the toggle-switch dynamic for a large variety of topological alterations

The TI system shows robust switching, as the system presents a wide bistability region (Fig. 2b). It is well-known that double-site phosphorylation systems, which involve the action of kinases and phosphatases, can generate bistability [24–26]. A simple double-site modification motif on the MAPK activation process has been investigated in that respect [24]. The MAPK motif may look similar to the TI network at first sight, however, the mechanism and its implications greatly differ. The MAPK system relies on external kinases and phosphatases to produce a bistable response. This bistable regimen is the result of the sequestration of these external enzymes in substrate-enzyme complexes. On the contrary, the TI system achieves the bistability through autocatalytic conversion of the single molecule into the antagonistic enzymatic conformations. These conversions, and the produced bistable region, are controlled, but not driven by the external level of a phosphate donor.

The TI system has a flexible structure, as the two catalytic forms can be reached through two separate paths of phosphorylation (through OP or PO). Flexible structures, such as the topology of the TI network, may retain their dynamical behaviour despite detrimental alterations. That is, even if the conformations lose their catalytic and/or spontaneous activity, the network structure is robust enough to maintain the toggle-switch dynamics. Due to our interest in the properties of the network instead of the effects of individual parameters, a *dummy* set of parameters is chosen. All catalytic reaction rates are equal to the unit (1 AU); all spontaneous reaction rates are set at the same low value of 0.05 AU (see Methods for more details). When we systematically test TI for the loss of path reactions (a catalytic and its associated spontaneous reactions), we find that systems with the loss of any one of the 8 reaction paths preserve the toggle-switch dynamics (Additional file 1: Figure S1).

**Fig. 3:**
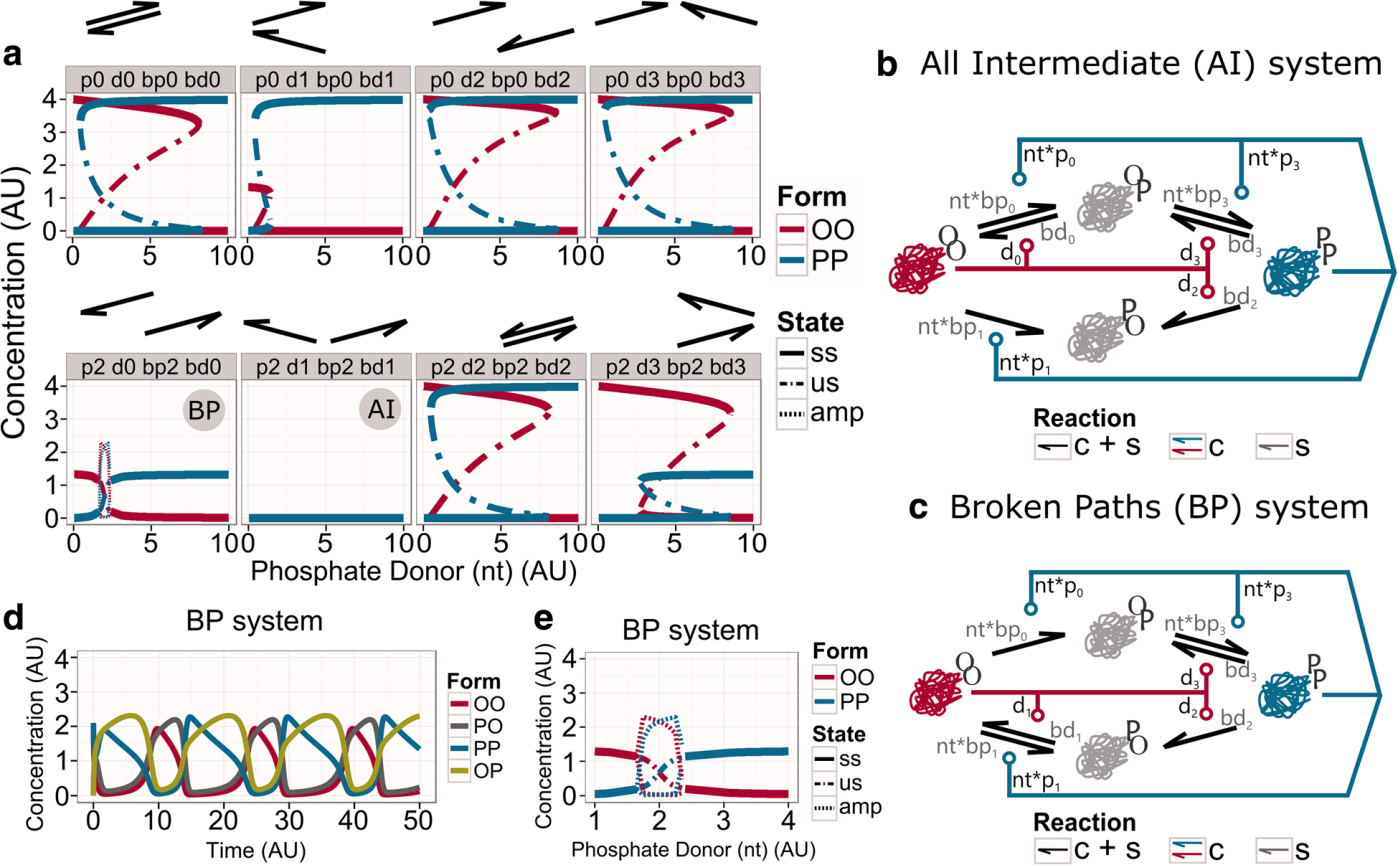
Analysis of removing reactions in pairs from the TI system. **a** Bifurcation diagrams showing the steady states of the systems where two catalytic reactions, and their corresponded spontaneous reactions, have been removed from the TI network. The arrows above each panel indicate which reactions have been dropped. The stable state (ss) is indicated by a solid line, the unstable steady state (us) is defined by the dash-dotted line, and the maxima and minima of oscillations (amp – for amplitude) are shown by dashed lines. **b** Wiring diagram of the All Intermediate (AI) network. The reactions that convert PO into OO and PP are both missing, so all molecules end up in this single-phosphorylated form. **c** Wiring diagram of the Broken Paths (BP) network. The reaction from PO to PP was dropped together with the reaction from OP to OO. **d** Time course diagram for the BP network in the oscillatory regime (phosphate donor *nt* = 2.2 AU). **e** Zoom in the bifurcation diagram of the BP system (panel “p2 d0 bp2 bd0”). See *networks topology* in Methods for more information about the structure. Table 2 in Methods contain the values of parameters

To show alternative dynamics, the TI network needs to suffer structural changes that affect at least two reaction paths (Fig. 3). There is a total of 16 different combinations when two reaction paths are lost (two catalytic and their spontaneous activities are removed). However, due to the symmetry of the TI network, all these systems can be represented by 8 topologies (Fig. 3a). Some of these networks show directionality for the conversions between OO and PP forms, but remarkably, the toggle-switch dynamic is preserved in 6 out of the 8 possible topologies (Fig. 3a). As we have previously mentioned, the symmetry of the topologies is extended to the parameter choice. Although the symmetry of the parameter set could potentially influence the bistable dynamics, random parameter perturbations in the AM system have only a minor effect on the switching dynamics [15].

There are two topologies, *All Intermediate* (AI) and *Broken Paths* (BP), that do not exhibit this behaviour (Fig. 3c, b). The AI network does not show any dynamical behaviour for changes in *nt* (Fig. 3a “p2 d1 pb2 bd1” panel, and 3b). In this topology, the abilities to change one of the single-phosphorylated forms (PO) are lost. The PO form serves now as a sink, and eventually, all molecules end up in this single-modified form (Fig. 3b). The BP network loses the bistability, although it keeps a sigmoid response for changes in *nt*. Strikingly, at the inflaction of this curve, the system shows a small region of oscillations (Fig. 3a “p2 d0 bp2 bd0” panel, and 3c). In this topology, the ability to add phosphates to one single-phosphorylated form (PO) is lost, together with the ability to remove phosphates from the other single-phosphorylated form (OP) (Fig. 3c). Thus, BP is a topology that works with directionality: PP can be reached only via OP, and OO can be reached via PO. The OO and OP forms are in balance with each other at low *nt* levels. This produces a reduction of the steady-state concentration of OO, compared with other topologies (Fig. 3a). When the phosphate donor increases beyond the threshold, a balance between PP and PO forms take control. Thus, together with the loss of hysteresis, the catalytic forms find the equilibrium at lower concentrations. Despite the loss of these dynamical features, over a small range of phosphate donor values (nt = [1.65, 2.40]), the BP network introduces a new behaviour: the oscillatory dynamics.

### Alterations may lead the toggle-switch behaviour into oscillatory dynamics

The emergence of oscillations in such a minimal system (BP system) could raise new, more complex responses. Oscillations enable biological systems to anticipate regular changes, either internal or external, and prepare the processes accordingly [27–29]. The simplest example is the circadian clock [22, 23, 30–34]. Biological networks produce two types of sustained oscillations, relaxation or delay oscillators [1, 26, 35–38]. Delay oscillators are composed of at least three components, contain at least a negative feedback loop (an odd number of negative interactions) and generate some sort of delay. Relaxation oscillators are composed of at least two components, containing negative and positive feedback loops. Based on these criteria the BP system works as a relaxation oscillator (Fig. 3d). In this type of oscillator, the concentration of molecules slowly changes over a long period of time, and abruptly varies in a short time [35, 36, 38].

Depending on the phosphate donor level, the BP network can show both switch-like and oscillatory behaviours. However, this system is not efficient in either of these. When it is working as an oscillator, this dynamic is limited to a small range of phosphate donor values (nt = [1.65, 2.40] AU) and it does not even achieve the maximal amplitude (Fig. 3d). This reduction occurs because the PP and OP forms are together at the same level, as the OO and PO forms are appearing at the same time (Fig. 3a, d). When we look at a wider range of phosphate donor (nt) values, we can observe that it works as a simple switch, without hysteresis. For the same reasons as above, the catalytic states cannot convert all molecules into their own form (Fig. 3a).

**Fig. 4:**
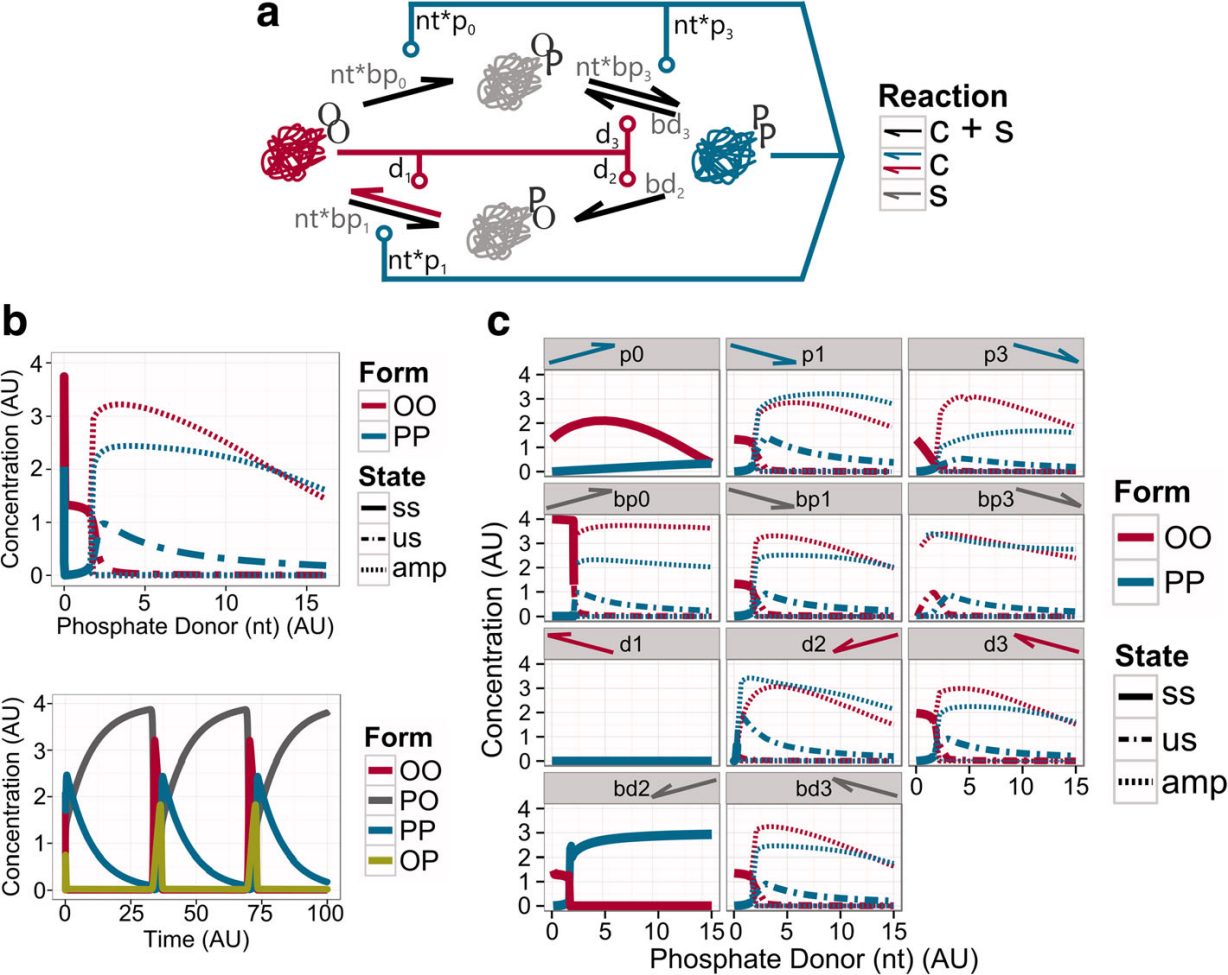
BD1 system and the behaviour of altered topologies diverged from it. **a** Wiring diagram of the BD1 network. **b** Bifurcation (up) and time course (down) analysis of the BD1 network. The bifurcation diagram shows the changes in the steady states of the catalytic forms. The stable steady state (ss) is indicated by a straight line, the unstable steady state (us) is defined by the dashed-dot line, and the maxima and minima of oscillations (amp for amplitude) are shown by dashed lines. The phosphate donor for the time course diagram is set to nt = 4 AU. **c** Bifurcation analysis of the effect of the loss of one extra reaction from the BD1 network. The legend above each panel indicates which parameters have been removed (by name of the parameter and the arrow that represents it). Labels same as on panel (**b**)

Thus, the BP network is not able to work as an efficient molecular sensor. If we assume that TI is an ancient molecular sensor, the modifications that lead to BP could have destroyed its original function, driving this system to disappear from ancient organisms. However, the appearance of the oscillatory behaviour might contribute to further explorations of new topologies in favour of keeping and improving this oscillatory dynamics, and exploit it for other functions (Fig. 4, Additional file 2: Figure S2). In this scenario, the BP network may have suffered further structural alterations, removing spontaneous and/or catalytic reactions to achieve a system with robust oscillatory dynamics (Fig. 4, Additional file 2: Figure S2). Strikingly, it is enough to drop the spontaneous dephosphorylation of PO into OO (bd1 parameter), to greatly enlarge the oscillatory region for changes in phosphate donor levels (nt = [1.60, 52.35]; Fig. 4b, Additional file 2: Figure S2).

This system (BD1 network) shows prominent relaxation-type of oscillations (Fig. 4b). In the BD1 system, PO behaves as a substrate, which slowly increases through the slow conversion from the PP form (since OO is almost totally absent, no autocatalysis helps this). When PO reaches a critical level, enough OO form appears to turn the autocatalytic steps on, converting eventually everything into OO. This form is not stable, so it is quickly converted back to PP through OP, and the whole process of building up PO starts from the beginning. This behaviour is maintained until high phosphate donor levels (nt = 52.35) are achieved. From this level, the oscillations are lost, since OO is getting totally absent and all molecules end up in the PO form (Fig. 4b, Additional file 2: Figure S2).

As shown above, the TI network can serve as a robust biological switch. It is resistant to perturbations in the form of loss of reaction paths in the network (Fig. 3a). Similarly, the BD1 network can preserve the oscillatory dynamic despite of various removals of catalytic and spontaneous reactions (Fig. 4c). Remarkably, the removal of three specific reactions leads to loss of the oscillatory behaviour. Both, the loss of the catalytic dephosphorylation of PO into OO (d1) and the catalytic phosphorylation of OO into OP (p0) (Fig. 4c), cut the remaining paths that can covert PP to OO and vice-versa. The loss of the spontaneous dephosphorylation of PP into PO (bd2) leads to a tiny regimen of oscillations (nt = [1.6–1.8] (AU)) before reaching an equilibrium (Table 1), where most molecules are converted into the double phosphorylated form (PP), while some goes to the single-modified form PO.

**Table 1:** Dynamical regimens of phosphate donor (nt) for each model. Columns indicate the type of behaviour; rows the models

	Bistable region	Oscillator
AM	0.50–8.55	–
TI	0.50–8.10	–
BP	–	1.65–2.40
BD1	–	1.60–52.35
CO	–	2.0 <
SO	–	1.9 <

### Minimal oscillatory topologies derived from the TI system

**Fig. 5:**
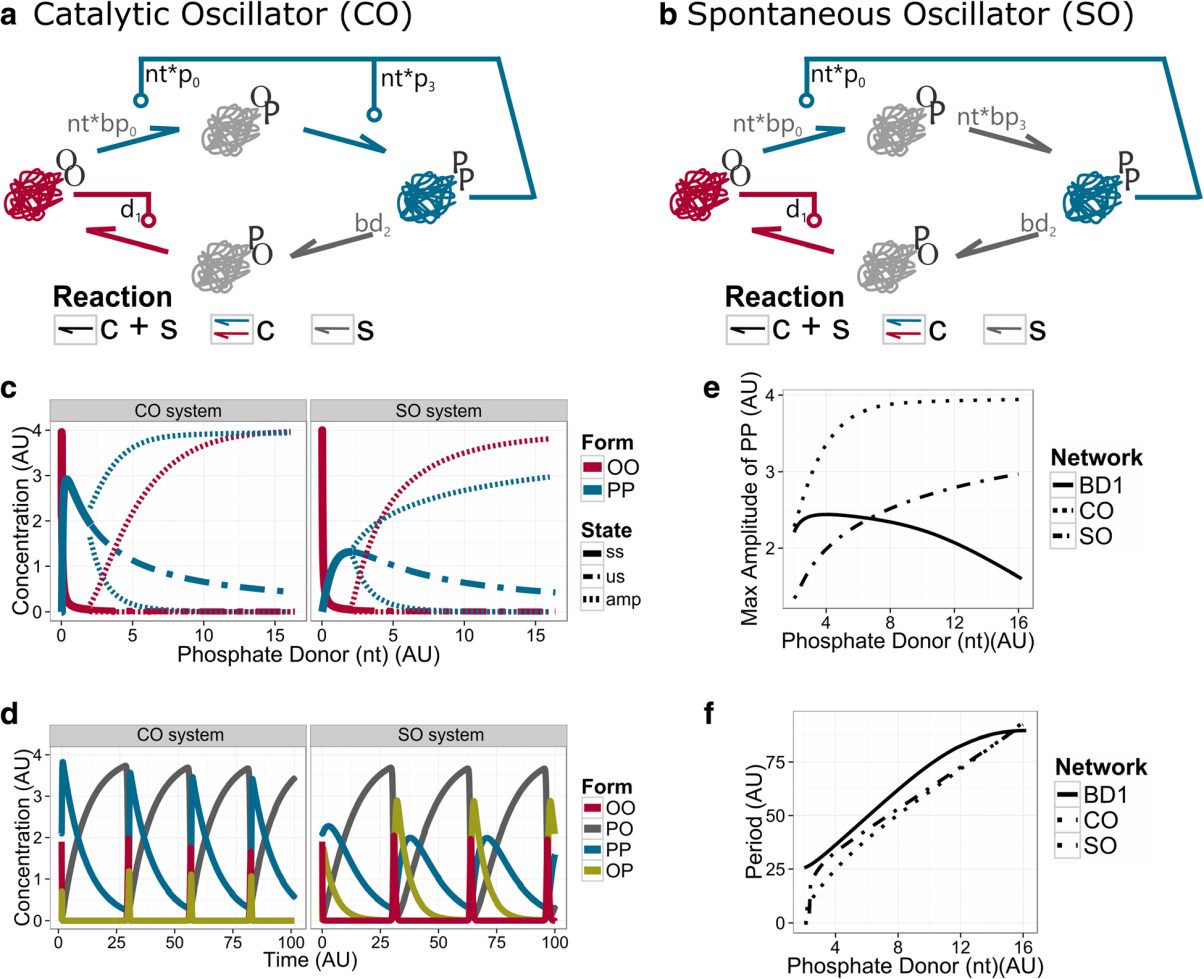
The minimal oscillatory systems: Catalytic Oscillator (CO) network and Spontaneous Oscillator (SO) networks. Wiring diagram of CO (**a**) and SO (**b**) networks. Both networks maintain the spontaneous dephosphorylation of PP into PO (grey arrow), OO has the control over the modification of PO into itself, and PP keeps its catalytic control over the transition from OO to OP. In the CO network PP also catalyses the phosphorylation of OP into PP, while in the SO network this reaction is spontaneous. **c** Bifurcation analysis. The graphic shows how the steady-state concentration of the catalytic molecules, OO and PP are affected by the availability of phosphate donor (nt). Stable steady states (ss) precedes the oscillatory solutions (unstable steady state (us) – dashed line, max and min of the amplitude of oscillation (amp) - striped lines). **d** Time course diagram. The phosphate donor is set to *nt* = 4 AU. **e** Maximal amplitude of the BD1, CO and SO networks. **f** Period of the BD1, CO and SO networks over a range of phosphate donor values. See *networks topology* in Methods for more information about the structure and Table 2 in Methods for values of parameters

Interestingly, other reductions of the BD1 network can lead to minimal systems, preserving the essential reactions for oscillatory dynamics (Fig. 5). The smallest topologies maintain the key reactions, which could not be removed from BD1 (Fig. 4): [1] the catalytic phosphorylation of OO into OP (p0), [2] the catalytic dephosphorylation of PO into OO (d1), and [3] the spontaneous dephosphorylation of PP into PO (bd2). To close the loop and reach all conformations, the phosphorylation of OP into PP can be driven either by the catalytic (*Catalytic Oscillator* (CO) network) or by the spontaneous reactions (*Spontaneous Oscillator* (SO) network) (Fig. 5a, b).

CO and SO networks work as relaxation oscillators (Fig. 5d). Similar to the BD1 network, the PO form works as a substrate and antagonises the catalytic forms OO and PP. In contrast to the BD1 network, the amplitudes of the catalytic forms of the smallest topologies increase with the phosphate donor (Fig. 5e). The CO system reaches the upper bound of amplitude faster than the SO system, merely because in the CO system PP catalyses the phosphorylation of OP, instead of leaving it for spontaneous conversion. For most of the oscillatory period, the molecules are slowly converted back to PO from the PP form. Once PO is leaking through OO, OO form turns on its autocatalysis, and converts all PO into OO. Then, OO is quickly converted back to PP. In the SO system we see that OP appears temporally, while in the CO system, the autocatalysis by PP is too fast to allow the appearance of high levels of OP. Despite this difference, both systems run with similar periods of oscillation (Fig. 5f). These two networks can be considered as minimal single molecule oscillators. Below, we discuss their possible relevance as timekeeping regulatory networks. Note, that the CO and SO networks can be converted symmetrically to systems where OO and PP are reverted.

## Discussion

Various evolutionary theories try to explain the appearance of complex regulatory interactions [39–41]. But it is generally proposed that the emergence of self-replicators and autocatalytic proteins was a key step in this [42, 43]. Following this idea, we introduced the TI model as a biologically feasible version of the Approximate Majority algorithm [16] as a minimal autocatalytic biological switch.

We could not identify an exact biological example for the TI system. We would require a single molecule that can take two opposing catalytic activities, and modify itself by these catalytic reactions. There are several systems which show similar network topologies and dynamics [17], but in these cases the feedback loops are not direct, instead intermediate molecules are affecting the catalytic reactions. Still, it is tempting to consider TI as a topology that could have served as a molecular sensor in primitive chemical or biological systems. Most biological processes are controlled by the energy state of cells [44]. As the TI network uses high energy nucleotides to convert itself between various states, it could have been acting as a nucleotide sensor in ancient systems. Particularly, TI would be found in PP conformation when the energy in the system is high, and in OO conformation when that energy is low (Fig. 2a,b). Since TI can robustly keep this feature (Fig. 3), one of the derivatives of TI could have been enough to serve as such a molecular sensor. In fact, the AM network is a culmination of the reduction, as this topology just preserves a single path for phosphorylation and dephosphorylation (Fig. 1a).

The energy state of a biological system is closely linked to the environmental conditions that surround it. As an example of this, photosynthetic organisms have a higher energy level during daylight, while reducing it in dark conditions [31, 34]. If the TI network would be used by such a cell, then it would be in the PP conformation during the daytime, and in an OO conformation at night, following the changes in ATP availability. As a function of light conditions, these alternating states of the TI network could affect internal processes. Since further loss of reaction paths can convert TI into a minimal oscillators such as BD1 (Fig. 4), CO or SO (Fig. 5), this could have helped the evolution of primitive circadian clocks that can anticipate changes in light conditions [45].

The minimal biological oscillator, called the KaiABC system controls the circadian clock in cyanobacteria [30, 46]. Importantly, the KaiABC-system generates sustained autonomous oscillations in vitro, when the three purified proteins, KaiC, KaiB and KaiA, and adenosine triphosphate (ATP) are mixed together [30]. In vivo, specific proteins regulating molecular activities during the day or night are binding to distinct forms of KaiC, the central molecule of the system [32, 33]. KaiC contains two phosphorylation sites (T432 and S431; abbreviated as T and S). During a 24 h period, it goes through a specific path of phosphorylation status changes: ST - > S/pT - > pS/pT - > pS/T - > ST (where p labels if a site is phosphorylated). The phosphorylation status of KaiC is the main driver of the oscillatory behaviour, and also the indicator of which processes (day or night) will be activated [47]. During daylight, KaiC autophosphorylates, and during the night, KaiC dephosphorylates itself using the same active site. The autokinase activity of KaiC is enhanced by KaiA, and the autophosphatase activity enhanced by KaiB [48, 49].

**Fig. 6:**
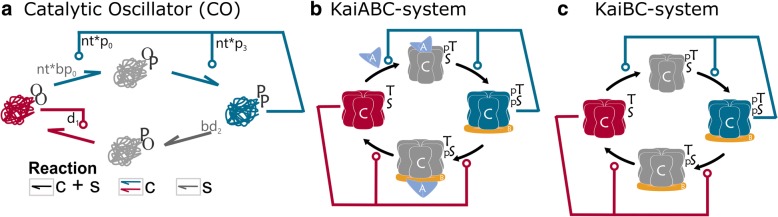
Similarities in the topologies of the CO oscillatory network and the KaiC system of cyanobacteria. **a** CO system (**b**) KaiABC-system of *S. elongatus*. KaiA (blue triangle) stimulates the autokinase activity of KaiC to covert the fully dephosphorylated form of KaiC (red) into its fully phosphorylated form (blue). KaiB (flat orange circle) binds to fully phosphorylated KaiC, this leads to the displacement of KaiA and enhances the autophosphatase activity of KaiC. **c** KaiBC-system of *Prochlorococcus*. KaiB (flat orange circle) binds KaiC in its fully phosphorylated form (blue) and enhances its autophosphatase activity, but KaiA is missing, so KaiC can phosphorylate itself without support from KaiA [61–64]

Figure 6 shows that the topology of the KaiABC-system shows high similarity to the above proposed minimal systems derived from TI. All the minimal models contain a single type of molecule with two sensitive sites of modification, four different conformations, and a phosphate donor is acting as the driver of the modifications. However, the dynamical behaviour of TI and most of its single reaction loss derivatives show bistability, not oscillations (Fig. 3). Stable relaxation oscillatory dynamics appears only for further reductions of the system (Fig. 3).

Although the topology of the KaiABC-system resembles the minimal oscillatory systems CO and SO, there is a major difference between them. The derivate systems from the TI network do not require auxiliary proteins to work as oscillators. In contrast, the autoregulation of KaiC requires KaiA and KaiB to run together the circadian clock of the cyanobacteria *Synechococcus elongatus* [48, 49]. The presence of external molecules has an impact on the type of oscillatory behaviour that the systems show. While the CO and SO models show relaxation-type of oscillations, experiments and proposed models of the KaiABC system show sinusoid-like oscillations [50–52]. Similarly, the small oscillatory regime of the more complex BP system above shows more sinusoid-like oscillation (Fig. 3d). It has also been proposed that the core of the cyanobacteria circadian clock works as a relaxation oscillator [53, 54], which better match to the results of the minimal CO and SO models. Sinusoid-like oscillators can arise from a relaxation oscillator by adding an extra negative feedback loop, causing the delay that leads to a more symmetric oscillation. This external negative feedback loop comes through the interaction of external molecules KaiB and KaiC in cyanobacteria, and if similar effects are added to our CO and SO models we can also convert them to show more symmetric oscillations (Additional file 3: Figure S3).

Homologs of KaiC and KaiB have been found in various bacteria and archaea species [55–59]. Where KaiA is missing, the KaiC homologs show alterations in their sequences, which might facilitate their autophosphorylation reactions. These systems are closer to the proposed CO system, although here KaiB is still required for proper dephosphorylation steps (Fig. 6). The KaiBC system can sustain the circadian oscillations in purple bacteria [60], but in the marine cyanobacteria *Prochlorococcus* species, this system shows an hourglass behaviour. In these systems, the phosphorylation autonomously runs in one direction, but it requires a daily reset induced by environmental triggers [61–64]. There are organisms where both KaiA and KaiB are missing, while KaiC is still present [65]; but it is still not clear what related dynamics, if any, KaiC shows in these species [66–68]. Following our models, KaiC alone could exhibit either switch-like dynamics and work as a molecular sensor, like TI or one of its divergent topologies, or it could also show oscillatory dynamics, like CO or SO. The presented transition from TI towards CO and SO shows how a biological switch can be converted into an oscillator. This might have been a path the KaiC system followed; it could have started as a sensor of nucleotide level, then turned into an oscillator, when it could predict in advance the time when environment changes are expected [45].

Single molecule sensors of energy levels might have been required even at earlier stages of evolution. In a possible RNA world, riboswitches could have sensed various metabolites [69]. Riboswitches that are also ribozymes could have adjusted their self-regulatory rates dependent on the level of surrounding nucleotides [70]. In that way, these RNA molecules could have worked like the proposed TI network (Fig. 2) or one of its derivatives (Fig. 3) to sense nucleotide levels. This could have helped the emergence of replicating RNA molecules [71], which could have adjusted the initiation of their replication to the presence or absence of free nucleotides. These ideas sound theoretical, and indeed evolution could have taken different paths, but TI-like, robust RNA based bio-switches could be readily built [72], and probably these could be further modified to work as CO-like oscillators. Such oscillators could have been usefully in predicting daily fluctuations in energy resources, leading to the emergence of primitive circadian clocks with similar network structure as the one currently running (by proteins) in cyanobacteria. Similar, ATP dependent regulation of the cell cycle oscillations have also been observed recently [73, 74] further highlighting that ATP sensing based control of periodic processes could be highly beneficial and evolution could have selected for the appearance of such systems.

## Conclusions

We have shown that a single molecule with two autocatalytic conformations can work both as a biological switch and as an oscillator. Such an oscillator is driving the circadian clock in cyanobacteria. The proposed network could be the simplest biologically plausible system that could perform these functions. In a proposed realisation as a phosphorylation-dephosphorylation switch it could also serve as a nucleotide or energy sensor. Based on these findings we propose that such a molecule could have served as a cornerstone during the emergence of self-replicating complex systems.

## Methods

### Networks topology

The topologies (wiring diagrams) represent a chemical reaction network. The biological entities that interact to produce the different models are described as proteins. The AM system is composed by a single protein that can be found in three different conformations, called OO, OP and PP. The TI and its derived systems are also based on the same single molecule. However, the proteins in these systems can adopt an extra conformation, the PO conformation.

This single molecular species is a protein regulated through (de)phosphorylation events. Because these single molecules are modelled assuming only two phosphosites, the impact of the (de)phosphorylation events is interpreted as following: the conformation OO denotes that the protein has not been modified, or it has been double dephosphorylated; the OP and PO conformations indicate a single (de)phosphorylation event; and the PP state shows the effects of being double phosphorylated. Note that the letter *P* indicates which phosphosite is phosphorylated, while the letter *O* shows the phosphosite that is not modified.

In the graphical representations, the different conformations of the main molecule (OO, OP, PO and PP) are treated as distinct chemical species. Interactions between conformations are represented by directional arrows, which represent the direction of the conversion reactions. As the reactions can be spontaneous and/or catalysed, the colour of the arrows indicates the mechanism of conversion. Black arrows indicate that the reaction happens either through the spontaneous or the catalytic mechanism. Grey arrows present the reactions that only occur spontaneously, while coloured arrows (either red or blue) describe the reactions that only happen through catalysis.

### Modelling framework

The biological scenario developed in the main text is modelled under the concept of distributive step-modifications [75]. The distributive step-modification denotes the process by which an entity, that presents several sensitive sites for modification, takes at most one modification during a single encounter. This mechanism is explicit in the mathematical description when it is formulated following the mass action law. For example, a protein in the double dephosphorylated conformation OO adopts the double phosphorylated conformation PP after two separated modification encounters. This two step-modifications are defined separately through the mass action law, which is translated into a Hill-type dynamics of coefficient *n* = 2 [55].

The phosphorylation events modify the structure of the protein, and they may also change the function [105, 106]. Under this biological premise, the different conformations show the roles that proteins take in the systems. Concretely, the double (de)phosphorylated conformations OO and PP hold opposite catalytic activities. The OP and PO conformations are intermediary conformations that serve as a bridge between the catalytic conformations OO and PP. The existence of these intermediary conformations is key for modelling distributive step-modification processes. These conformations are the ones that introduce nonlinearity on the models.

The conversion between conformations may occur through two different mechanisms. A conformation undergoes either a spontaneous transformation, or a catalytic driven reaction. The spontaneous transformation is referred to a (de)phosphorylation event that the own molecule promotes. In the catalytic reaction, this (de)phosphorylation event is driven by one of the catalytic conformations, either OO or PP. These different mechanisms are included in the mathematical models through different terms in the quantitative descriptions. The catalytic driven terms include the catalyst and the substrate, while the spontaneous terms only contain the molecular species that changes.

The AM and TI systems are composed by either three or four conformations, respectively. However, the mathematically definition of one of the conformation is omitted in the quantitative models. Particularly, the missed equation is the one that details the behaviour of the intermediary conformation OP, which can be algebraically calculated, following the assumption that the total concentration of all the forms (*tot*) is fixed. In the case of the AM system this is: *OP = tot - PP - OO*. The remaining differential equations are:

1 $$ \frac{\mathrm{d} OO}{\mathrm{d}t}=d0\ast nd\ast OO\ast \left( tot- PP- OO\right)-p0\ast nt\ast PP\ast OO- bp0\ast nt\ast OO+ bd0\ast nd\ast \left( tot- PP- OO\right) $$

2 $$ \frac{\mathrm{d} PP}{\mathrm{d}t}=p3\ast nt\ast PP\ast \left( tot- PP- OO\right)-d3\ast nd\ast OO\ast PP+ bp3\ast nt\ast \left( tot- PP- OO\right)- bd3\ast nd\ast P $$

This approach is taken in order to ensure that the numerical simulations will be within the limits of the desired molecular concentration. The mathematical system of equations that describes the TI system and its derivatives also replaces the equation of the OP form for the subtraction *OP = tot - PP - OO – PO*:

3 $$ \frac{\mathrm{d} OO}{\mathrm{d}t}=-p0\ast nt\ast PP\ast OO-p1\ast nt\ast PP\ast OO+d0\ast nd\ast OO\ast \left( tot- PP- OO- PO\right)+d1\ast nd\ast OO\ast PO- bp0\ast nt\ast OO- bp1\ast nt\ast OO+ bd0\ast nd\ast \left( tot- PP- OO- PO\right)+ bd1\ast nd\ast PO $$

4 $$ \frac{\mathrm{d} PP}{\mathrm{d}t}=p3\ast nt\ast PP\ast \left( tot- PP- OO- PO\right)+p2\ast nt\ast PP\ast PO-d2\ast nd\ast OO\ast PP-d3\ast nd\ast OO\ast PP+ bp3\ast nt\ast \left( tot- PP- OO- PO\right)+ bp2\ast nt\ast PO- bd2\ast nd\ast PP- bd3\ast nd\ast PP $$

5 $$ \frac{\mathrm{d} PO}{\mathrm{d}t}=p1\ast nt\ast PP\ast OO-p2\ast nt\ast PP\ast PO+d2\ast nd\ast OO\ast PP-d1\ast nd\ast OO\ast PO+ bp1\ast nt\ast OO- bp2\ast nt\ast PO+ bd2\ast nd\ast PP- bd1\ast nd\ast PO $$

In both systems of equations, all terms are preceded by two parameters, the reaction rate and the modifier rate that modulates the propensity of the reaction. The reaction rates identify the type of reaction, spontaneous or catalytic, and its direction, towards OO or PP. Thus, spontaneous reactions that move to OO forms are indicated by bd_0–3_, while if it moves to PP forms, the parameters bp_0–3_ are used. The catalytic reactions that go towards OO are described by d_0–3_, and the ones that go towards PP are defined by the parameters p_0–3_. As catalytic reactions are faster, the reaction rates of spontaneous reactions are lower than the rates of the reactions that are catalysed.

The modifier rate identifies if the modification event is either a dephosphorylation or phosphorylation case. Phosphorylation events add a phosphate group from a phosphate donor in a sensitive site, while dephosphorylation events remove these phosphate groups and add them to a phosphate acceptor. The level of phosphate donor and acceptor are represented by the *nt* and *nd* parameters, respectively. The *nt* parameter is included in all spontaneous and catalytic reactions that move towards PP, while the *nd* parameter is contained in all spontaneous and catalytic reactions that go towards OO. In a biological context, the availability of the phosphate donor is usually more limited than the acceptor. Thus, the *nd* parameter stays as a fixed threshold in all models, while the *nt* parameter varies over a determined range. The specific values for each parameter previously described can be found in Table 2.

**Table 2:** Values of the parameters to conduct bifurcation analysis. Columns are the networks, and rows are the parameters. Initial conditions do not matter for these calculations, for simulations of oscillations we set *OP* = *PO* = 0 and *OO* = 2.1 *PP* = 1.9 to start from an asymmetric state

	AM	TI	BP	BD1	CO	SO
tot	4.0	4.0	4.0	4.0	4.0	4.0
nt	[0.0,10.0]	[0.0,10.0]	[0.0,10.0]	[0.0,15]	[0.0,15]	[0.0,15]
nd	2.0	2.0	2.0	2.0	2.0	2.0
p0	1.0	1.0	1.0	1.0	1.0	1.0
p1	–	1.0	1.0	1.0	–	–
p2	–	1.0	–	–	–	–
p3	1.0	1.0	1.0	1.0	1.0	–
d0	1.0	1.0	–	–	–	–
d1	–	1.0	1.0	1.0	1.0	1.0
d2	–	1.0	1.0	1.0	–	–
d3	1.0	1.0	1.0	1.0	–	–
bp0	0.05	0.05	0.05	0.05	–	–
bp1	–	0.05	0.05	0.05	–	–
bp2	–	0.05	–	–	–	–
bp3	0.05	0.05	0.05	0.05	–	0.05
bd0	0.05	0.05	–	–	–	–
bd1	–	0.05	0.05	–	–	–
bd2	–	0.05	0.05	0.05	0.05	0.05
bd3	0.05	0.05	0.05	0.05	–	–

### Bifurcation and time-course analysis

The bifurcation diagrams presented here (Figs. 1b; 2b; 3a; 4c; 5c) show the steady-state behaviour of the enzymatic forms OO and PP as the phosphate donor (nt) varies. The time-course diagrams (Figs. 3d; 4b; 5d) present the concentrations of all molecules as a function of time for a certain value of phosphate donor (nt).

All time-course and bifurcation analysis are obtained through our methods developed in R (version 3.2.2) [76]. These methods exploit the functions provided in deSolve [77] and rootSolve packages [78, 79] to conduct the numerical solutions. The methods to conduct these analyses are available for use at https://github.com/rosherbal/sw2os. The graphical representation of the analysis make use of the ggplot2 package [80]. Functions from the reshape2 package [81] allow to obtain an appropriate format for plotting the data with ggplot2.

The models presented here were previously tested on commercial software. These tools are Visual GEC (http://biology.azurewebsites.net/gec/) and Oscill8 (http://oscill8.sourceforge.net/).

## Additional files

Additional file 1: **Figure S1.** Removal of reactions from the TI network. a) Bifurcation diagrams showing the steady states of the systems where a reaction path is lost from the TI network. The arrows above each panel indicate which reactions have been dropped from the TI network. The name of the reaction rates that are dropped are also indicated above each panel. The stable state (ss) is indicated by a straight line, while the unstable state (us) is defined by the dashed-dot line. b) The wiring diagram of the Two Intermediates (TI) system. (PDF 285 kb)

Additional file 2: **Figure S2.** Removal of reactions from the BP network. a) Bifurcation diagrams showing the steady states of the systems where either a catalytic or a spontaneous reaction have been removed from the BP network. The arrows above each panel indicate which reactions have been dropped from the BP network. The name of the reaction rates that are dropped are also indicated above each panel. The stable state (ss) is indicated by a straight line, the unstable state (us) is defined by the dashed-dot line, and the maximal and minimal amplitudes (ma) are shown by the striped lines. b) Broken Paths (BP) system. (PDF 313 kb)

Additional file 3: **Figure S3.** Negative feedback driven oscillations of a TI derivative system. a) Wiring diagram of a TI system derivate that interacts with an external molecule that presents two conformations (A and B). b) List of reactions. The left column shows the catalytic reactions driven by the main TI system, while the right column presents the reactions established with the external conformations A and B. On top of each reaction arrow, the parameter names that affect the specific reactions are indicated. c) Time-course diagram showing the sinusoidal behaviour of the system. Initial concentrations of the molecules: *OO* = 2; *PP* = 1; *B* = 2 AU, all others are 0. Parameters: *p*_*0*_ = 1, *p*_*3*_ = 0.5, *d*_*1*_ = 0.5, *d*_*2*_ = 1, *k*_*3*_ = 0.1, *tot* = 3. (PDF 390 kb)

## Abbreviations

AI: All Intermediate
AM: Approximate Majority
BP: Broken Paths
CL: Critical level
CO: Catalytic oscillator
nd: Phosphate acceptor
NDP: Nucleotide diphosphate
nt: Phosphate donor
NTP: Nucleotide triphosphate
SO: Spontaneous oscillator
TI: Two intermediates
