# Supplementary material for: Single molecules can operate as primitive biological sensors, switches and oscillators
Source: BMC Syst Biol. 2018 Jun 18;12:70. doi: 10.1186/s12918-018-0596-4 (PMC6007071; doi:10.1186/s12918-018-0596-4)
Supplement: Supplementary file 1 — Figure S1. Removal of reactions from the TI network. a) Bifurcation diagrams showing the steady states of the systems where a reaction path is lost from the TI network. The arrows above each panel indicate which reactions have been dropped from the TI network. The name of the reaction rates that are dropped are also indicated above each panel. The stable state (ss) is indicated by a straight line, while the unstable state (us) is defined by the dashed-dot line. b) The wiring diagram of the Two Intermediates (TI) system. (PDF 285 kb) [file 12918_2018_596_MOESM1_ESM.pdf]

Single molecules can operate as primitive biological sensors, switches and oscillators

Rosa D. Hernansaiz-Ballesteros[1], Luca Cardelli[2,3] & Attila Csikász-Nagy[1,4]

[1] Randall Centre for Cell and Molecular Biophysics and Institute for Mathematical and Molecular Biomedicine, King's College London, London, SE1 1UL f
[2] Microsoft Research, 21 Station Road, Cambridge CB1 2FB, UK
[3] Department of Computer Science, University of Oxford, Wolfson Building, Parks Road, Oxford OX1 3QD, UK
[4] Faculty of Information Technology and Bionics, Pázmány Péter Catholic University, H-1083 Budapest, Hungary

Supplementary Figure S1

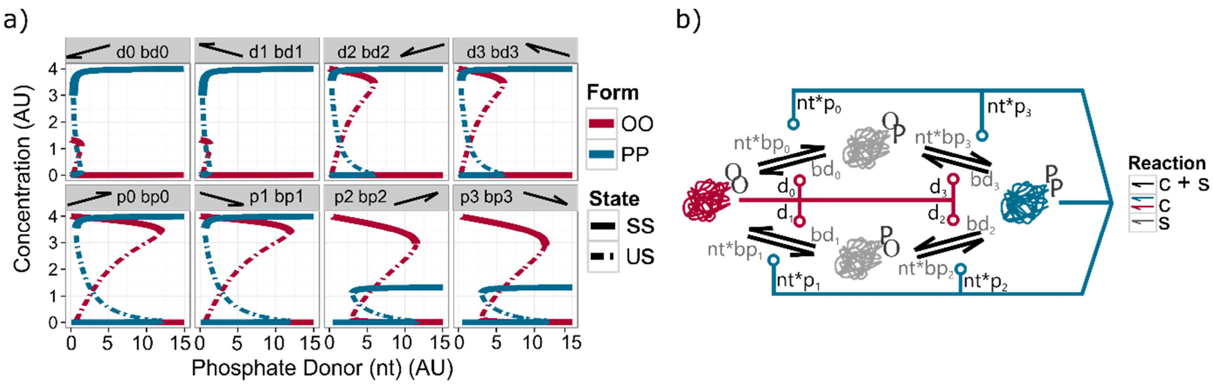

Figure S1. Removal of reactions from the TI network. a) Bifurcation diagrams showing the steady states of the systems where a reaction path is lost from the TI network. The arrows above each panel indicate which reactions have been dropped from the TI network. The name of the reaction rates that are dropped are also indicated above each panel. The stable state (ss) is indicated by a straight line, while the unstable state (us) is defined by the dashed-dot line. **b)** The wiring diagram of the Two Intermediates (TI) system.
