# Supplementary material for: Single molecules can operate as primitive biological sensors, switches and oscillators
Source: BMC Syst Biol. 2018 Jun 18;12:70. doi: 10.1186/s12918-018-0596-4 (PMC6007071; doi:10.1186/s12918-018-0596-4)
Supplement: Supplementary file 3 — Figure S3. Negative feedback driven oscillations of a TI derivative system. a) Wiring diagram of a TI system derivate that interacts with an external molecule that presents two conformations (A and B). b) List of reactions. The left column shows the catalytic reactions driven by the main TI system, while the right column presents the reactions established with the external conformations A and B. On top of each reaction arrow, the parameter names that affect the specific reactions are indicated. c) Time-course diagram showing the sinusoidal behaviour of the system. Initial concentrations of the molecules: OO = 2; PP = 1; B = 2 AU, all others are 0. Parameters: p0 = 1, p3 = 0.5, d1 = 0.5, d2 = 1, k3 = 0.1, tot = 3. (PDF 390 kb) [file 12918_2018_596_MOESM3_ESM.pdf]

Single molecules can operate as primitive biological sensors, switches and oscillators

Rosa D. Hernansaiz-Ballesteros[1], Luca Cardelli[2,3] & Attila Csikász-Nagy[1,4]

[1] Randall Centre for Cell and Molecular Biophysics and Institute for Mathematical and Molecular Biomedicine, King's College London, London, SE1 1UL f
[2] Microsoft Research, 21 Station Road, Cambridge CB1 2FB, UK
[3] Department of Computer Science, University of Oxford, Wolfson Building, Parks Road, Oxford OX1 3QD, UK
[4] Faculty of Information Technology and Bionics, Pázmány Péter Catholic University, H-1083 Budapest, Hungary

Supplementary Figure S3

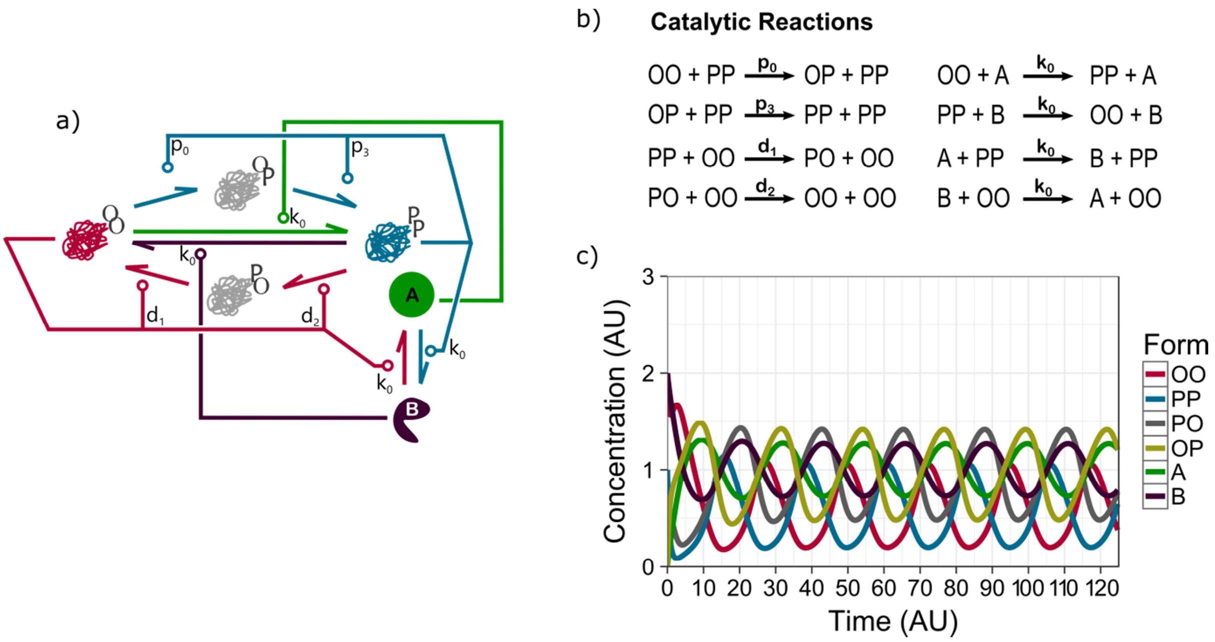

Figure S3. Negative feedback driven oscillations of a TI derivative system. a) Wiring diagram of a TI system derivate that interacts with an external molecule that presents two conformations (A and B). b) List of reactions. The left column shows the catalytic reactions driven by the main TI system, while the right column presents the reactions established with the external conformations A and B. On top of each reaction arrow, the parameter names that affect the specific reactions are indicated. c) Time-course diagram showing the sinusoidal behaviour of the system. Initial concentrations of the molecules: *OO* = 2; *PP* = 1; *B* = 2 AU, all others are 0. Parameters: p_0 = 1, p_3 = 0.5, d_1 = 0.5, d_2 = 1, k_3 = 0.1, *tot* = 3
